# Molecular identification of* Malassezia* species isolated from neonates hospitalized in Neonatal intensive care units and their mothers

**DOI:** 10.18502/cmm.7.3.7800

**Published:** 2021-09

**Authors:** Kamiar Zomorodian, Maryam Naderibeni, Hossein Mirhendi, Mostajab Razavi Nejad, Seyed Mojtaba Saneian, Mozhgan Mahmoodi, Mahboobeh Kharazi, Hossein Khodadadi, Keyvan Pakshir, Marjan Motamedi

**Affiliations:** 1 Department of Medical Parasitology and Mycology, Faculty of Medicine, Shiraz University of Medical Sciences, Shiraz, Iran; 2 Basic Sciences in Infectious Diseases Research Center, Shiraz University of Medical Sciences, Shiraz, Iran; 3 Department of Medical Parasitology and Mycology, Faculty of Medicine, Isfahan University of Medical Sciences, Isfahan, Iran; 4 Department of Pediatrics, Neonatal Research Center, Shiraz University of Medical Sciences, Shiraz, Iran

**Keywords:** Breastfeedings, * Malassezia*, *M. arunalokei*, Neonate, Sequencing

## Abstract

**Background and Purpose:**

Given the important role of* Malassezia* spp. in skin diseases and other associated infections in neonates, this study aimed to investigate the presence and frequency of* Malassezia* spp. in the skin of neonates hospitalized in neonatal intensive care units and their mothers using culture and accurate molecular-based methods.

**Materials and Methods:**

In total, 205 samples were collected from 130 neonates (>4-day-old) and 75 mothers. Isolation of* Malassezia* spp. from the skin was performed using Leeming-Notman agar and modified Dixon agar media. To compare the* Malassezia* microflora on the skin of the neonates and their mothers, a polymerase chain reaction-sequencing method was performed for spp. identification of 92 isolates obtained from neonates and their mothers. Moreover, possible associated risk factors for the colonization of* Malassezia* spp. on the skin were recorded.

**Results:**

Cultures from 62.3% of neonates and 77.3% of mothers were positive for* Malassezia* spp. growth. *Malassezia globosa* was the most prevalent isolated spp. found in the skin of the study population. It is noteworthy that a rare* Malassezia* spp., *Malassezia arunalokei*, was isolated from the skin of one neonate. There was a 76% similarity between the mother-neonate isolate sequences results. The statistical analysis showed that the type of feeding is a significant (*P*<0.001) associated factor for* Malassezia* skin colonization.

**Conclusion:**

The findings support the hypothesis that the colonization of* Malassezia* in neonates is significantly influenced by that of the mother, and this may be associated with breastfeeding.

## Introduction

Afetus Develops for approximately nine months in a microorganism-free environment and is subsequently delivered to an external area. Exposure of the neonate to a wide variety of microbes begins immediately at birth, and prematurity is the most important predisposing factor for neonatal infection during the first week after birth [ 1 ]. Neonates have no mycoflora at birth; the fungal infections can manifest as superficial mucocutaneous infections or life-threatening disseminated sepsis in neonates [ 2 ]. *Candida* and* Malassezia* are the two most frequent genus of opportunistic yeasts causing a variety of fungal infections in premature neonates [ 3 ]. 

*Malassezia* spp. are the predominant commensal yeasts harboring up to 80% of the microbiota of human skin [ 4 ]. These lipophilic yeasts are directly/indirectly associated with many skin diseases, such as pityriasis versicolor, seborrhoeic dermatitis,* Malassezia* folliculitis, psoriasis, and atopic dermatitis [ 5 ]. Aside from superficial skin infections associated with* Malassezia*, they are known as potential agents of fatal venous catheter-related fungemia in premature neonates, particularly in those receiving intravenous lipid emulsions [ 6 ]. As skin colonization may lead to catheter colonization, it is important to learn how neonates in an intensive care setting become colonized/infected with* Malassezia* spp. [ 4 ]. 

Although the source of* Malassezia* colonization in neonates is not clear enough, the kangaroo mother care, a method of caring for preterm neonates with skin-to-skin contact, may be a major commencement factor for* Malassezia* colonization on the skin of neonates. 

Given the importance of the ability of* Malassezia* spp. to cause skin diseases or other associated infections in neonates, this study aimed to assess the presence and frequency of* Malassezia* spp. on the skin of neonates hospitalized in neonatal intensive care units (NICUs) and their mothers.

## Materials and Methods

### Sample collection and fungal culture

This study was carried out from January 2019 to April 2020. The study protocol was cleared by the Research Ethics Committee of Shiraz University of Medical Sciences (IR.SUMS.REC.1398.204). This study was performed on 130 neonates (&gt;4-day-old) admitted to the NICU in Zeinabieh Hospital, Shiraz, Iran. Sample collection from the skin of neonates was performed on the first or fourth day after birth. Moreover, samples of mothers who gave birth at the same hospital were collected immediately after childbirth. Some neonates were born in another hospital but were admitted to this hospital to use the NICU facilities.

Demographic characteristics of the neonates, including gender and birth weight, were recorded, and putative risk factors responsible for skin colonization were noted as well. The putative risk factors variables were considered in the use of anti-bacterial and anti-fungal medication, birth weight, vascular catheterization, phototherapy, cause of hospitalization in NICU, type of feeding, breathing, and delivery.

The samples were collected after obtaining written informed parental consent. A complete skin examination was performed prior to sampling, and skin sample was collected using adhesive cellophane tape as follows; a 5-cm-long and 2-cm-wide scotch tape was applied on the chest, pressed firmly, and then removed. Subsequently, the tape was pasted on the surface of a glass slide. The scotch tape was cut into pieces of 1.5×2 cm by sterile scalpel and inoculated into plates containing Leeming-Notman agar medium (1% peptone, 0.5% glucose, 0.1% yeast extract, 0.4% desiccated ox bile, 0.1% glycerol, 0.05% glycerol monostearate, 0.05% Tween 60, 1% whole-fat cow's milk, and 1.5% agar). The plates were incubated at 30˚C for 2 weeks and checked for colony formation every day. The isolated colonies were then sub-cultured on modified Dixon agar (36 g malt extract, 6 g peptone, 20 g desiccated ox-bile, 10 mL Tween 40, 2 mL glycerol, 2 mL oleic acid, 12 g agar, 1 L distilled water, pH 6) to remove contamination and obtain maximum positive culture results. The modified Dixon agar culture plates were incubated for 4 weeks at 30 °C in a humid chamber. 

### Preparation of genomic DNA

For DNA extraction, the cells scraped from the modified Dixon agar media were transferred into 500 μL of a lysis buffer (containing 10 mM Tris-HCL [pH 8], 1 mM EDTA [pH 8], 100 mM NaCl, and 1% sodium dodecyl sulfate), kept for 30 min and crushed for 3 min by a mechanical grinder. Subsequently, the extracted DNA was purified by phenol chloroform-isoamyl alcohol as described by Gupta et al. [ 7 ]. 

### Polymerase chain reaction and sequencing of the D1/D2 region

Identification of all the isolates was performed by the amplification of approximately 580 bp polymerase chain reaction (PCR) product of D1/D2 region of small subunit 26S rDNA using the primers 5' TAACAAGGATTCCCCTAGTA and 5'ATTACGCCAGCATCCTAAG [ 8 ]. The PCR reactions were performed using 2X PCR premix (Amplicon, Denmark), 0.5 μM of each primer, 4 μL of DNA template, and sufficient distilled water to reach a final volume of 50 μL. The amplification was accomplished using one cycle at 94 ˚C for 5 min, 30 cycles of denaturation at 94 ˚C for 45 sec, annealing at 55˚C for 1 min, extension at 72 ˚C for 45 sec, and a final extension step at 72 ˚C for 7 min. The PCR products were evaluated by 1.1% (w/v) agarose gel electrophoresis onto Tris-Borate-EDTA buffer (Tris 90mM, Boric acid 90 mM, EDTA 2 mM) stained with 0.5 μg per ml ethidium bromide, and photographed under UV transillumination. A reference* Malassezia* isolate (GenBank accession number MT211520) was used as a positive control in each PCR reaction. The PCR results were validated only when the negative control samples with no DNA was demonstrated not amplicon after PCR. 

The positive PCR products were subjected to sequencing using the forward primer as for the PCR, via an automated DNA sequencer (ABI PrismTM 3500 Genetic Analyzer, Genetic Group). The sequences were edited with Geneious software (version 11.1), and the identity of the spp. was determined by comparing the obtained sequences with those reliable sequences deposited in the GenBank database (https://blast.ncbi.nlm.nih.gov/Blast).

### Statistical analysis

For categorical variables in 2×2 tables, if there was a cell with expected frequencies of less than five, Fisher’s exact test was used. However, if there was not such a cell, the Chi-squared test with continuity correction was used. It should be mentioned that a p-value of less than 0.05 was considered statistically significant. All statistical analyses were performed in SPSS statistical software (version 10.0, SPSS Inc, IBM).

## Results

In total, 130 neonates who were hospitalized in NICU were enrolled in this study, 66 (50.8%) and 64 (49.2%) of whom were male and female, respectively. In this study, the overall colonization rate was 62.3%. In total, 22 (45.8%) and 59 (72%) out of the 48 and 82 neonate samples collected from the first and fourth day of birth, respectively, were colonized with* Malassezia*. According to the results based on the culture method,* Malassezia* spp. were isolated from 58 out of 75 (77.3%) samples collected from mothers. 

To compare the skin* Malassezia* microflora of the neonates with those of their mothers, the results of the D1/D2 sequence were BLAST-analyzed for 46 neonatal samples and 46 maternal samples. Based on the sequencing analysis of* Malassezia* spp. isolated from 46 samples of neonates, *Malassezia globosa* was the most isolated spp. from the skin mycoflora of neonates (n=28, 60.9%), followed by* Malassezia restricta* (n=8, 17.4%), and* Malassezia furfur* (n=7, 15.2%),* Malassezia sympodialis* (n=2, 4.3%), and *Malassezia arunalokei* (n=1, 2.2%). 

Analysis of the 46 DNA sequences obtained from isolates recovered from mothers indicated that *M. globosa* was the predominant spp. (n=31, 67.4%) followed by *M. restricta* (n=7, 15.6%), *M. furfur* (n=7, 15.6%), and lastly, *M. sympodialis* (n=1, 2.2%). Representative sequences of the neonatal isolates and those of their mothers are deposited in the GenBank database with accession numbers MT211519-MT211538 and MW007921-MW007988. Moreover, the sequence of *M. arunalokei*, as a rare spp. isolated in this study, has been deposited in the Genbank database with the accession number of MW007946.

Generally, as shown in Table 1, there was a 76% similarity between the* Malassezia* spp. isolated from neonates and those isolated from their mothers. In the analysis of this relationship, a total of nine patterns of* Malassezia* spp. were seen between these two populations surveyed, the highest percentage of which belonged to *M. globosa* pattern with 54.3%.

**Table 1:** Correlation between the spectra of *Malassezia* species isolated from neonates and their mothers based on sequencing results

Pattern number	n (%)	*Malassezia* isolated from neonates	*Malassezia* isolated from mothers
1	25 (54.3%)	*M. globosa*	*M. globosa*
2	5 (10.8%)	*M. furfur*	*M. furfur*
3	4 (8.7%)	*M. restricta*	*M. restricta*
4	4 (8.7%)	*M. furfur*	*M. globosa*
5	2 (4.3%)	*M. globosa*	*M. sympodialis*
6	2 (4.3%)	*M. globosa*	*M. restricta*
7	2 (4.3%)	*M. furfur*	*M. restricta*
8	1 (2.1%)	*M. arunalokei*	*M. globosa*
9	1 (2.1%)	*M. sympodialis*	*M. restricta*
Total	46 (100%)		

Regarding the statistical analysis of the putative risk factors variables for* Malassezia* colonization in neonates during their stay in the NICU, there were no significant differences between* Malassezia* colonization in neonates and cause of hospitalization in NICU, type of breathing, type of delivery, birth weight, phototherapy, antibacterial therapy, antifungal therapy, and vascular catheterization. However, a significant relationship was observed between the type of feeding and acquisition of* Malassezia* skin colonization (*P*<0.001). In this regard, it was found that 58 out of the 81 neonates (71.6%) with* Malassezia* colonization were breast-fed. The univariate analysis of the risk factors associated with colonized and non-colonized neonates is shown in Table 2. 

**Table 2:** Univariate analysis of risk factors associated with colonization in neonates

Risk factor		colonization (81 cases)	no colonization (49 cases)	*P-value*
Cause of hospitalization	Premature	58	38	0.67
	Pneumonia	0	1	
	Septicemia	6	2	
	Mother's addiction	3		
	Other demonstrations	14	8	
Type of feeding	Breastfeeding	58	24	0.001
	Dry milk	11	4	
	Intravenous	12	21	
Type of breathing	Normal	49	26	0.23
	mechanical ventilation	32	23	
Type of delivery	Natural	16	13	0.39
	Cesarean	65	36	
Birth weight	1kg>	4	1	0.83
	1kg<	77	48	
Phototherapy	Received	22	8	0.19
	Not received	59	41	
Antibacterial treatment	Received	79	45	0.19
	Not received	2	4	
Antifungal treatment	Received	1	1	1.0
	Not received	80	48	
Vascular catheterization	Received	77	46	1.0
	Not received	4	3	

## Discussion

*Malassezia* fungemia, a rare difficult-to-control hospital-acquired infection, is one of the critical issues in the NICU. As evidenced by the outbreak investigations,* Malassezia* spp. firstly colonize the human skin and cause fungemia in susceptible individuals in later stages [ 9 , 10 ]. Different results with the declaration of no colonization to substantial colonization of* Malassezia* in the skin of neonates have been reported in previous literature [ 11 , 12 ]. According to this study, 62.3% of neonates acquired* Malassezia* spp. during their stay in the hospital, which shows almost a higher colonization rate, compared to previous studies [ 4 , 13 ]. The recovery rate of* Malassezia* spp. has been reported to be 0-68% during the first week of life in different studies [ 12 , 14 ]. 

The present study confirmed that the* Malassezia* skin colonization begins on the first day of life and develops significantly during the next days (45.8% colonization rate on the first day vs. 72% on the fourth day). Similar to other investigations, our results indicated a direct correlation between colonization rate and the stay duration in the hospital [ 11 , 15 ]. Contrary to the findings of this study, those of the studies conducted by Gupta et al. [ 4 ] and Ashbee et al. [ 12 ] revealed that none of the neonates were colonized on the first day of birth. In addition to the age of neonates at the time of sampling, this difference in colonization rates may be reflected in some variables, such as sample site (i.e., ear, forehead, trunk, cheek, and chest) and the studied neonatal population (healthy neonates or those admitted in the intensive care ward) [ 16 , 17 ]. 

Novel spp. identification methods, such as PCR, followed by sequencing of appropriate targets and obtaining data on genetic structure and variation of the fungal populations, have important implications for understanding the microbial epidemiology of the fungi. A limited number of studies have used molecular methods, such as real-time PCR [ 4 ] or PCR-restriction fragment length polymorphism [ 18 ], for spp. identification of* Malassezia* isolated from the skin of neonates. However, the majority of studies have identified the* Malassezia* spp. based on morphological and biochemical features [ 13 , 14 ]. To the best of our knowledge, this is the first study using the PCR-sequencing method to identify* Malassezia* spp. isolated from the skin of neonates.

Based on the findings of this study *M. globosa* was the most frequent in neonates. This is inconsistent with the results of other studies that have introduced *M. furfur* as the most common spp. of* Malassezia* skin mycoflora in neonates [ 4 , 14 ]. In the present study,* Malassezia obtuse*,* Malassezia slooffiae,* and* Malassezia japonicabased* were not found on sequencing the D1/D2 region of* Malassezia* genomic DNA. This was in contrast to the observation of Zomorodian et al. [ 18 ] or Ayhan et al. who reported the above-mentioned spp. among isolates [ 14 ]. 

Moreover, in the present research, a rare* Malassezia* spp., *M. arunalokei*, was isolated from the skin of a 4-day-old neonate. It is worth noting that Honnavar et al., in 2016, reported *M. arunalokei* for the first time during their survey on patients with seborrheic dermatitis and healthy individuals [ 19 ]. Similar to the present study, Gholami et al. reported *M. arunalokei* as the rare spp. of* Malassezia* from Iran; however, the strain was isolated from the scalp of a 23-year-old student [ 20 ]. 

The spectrum of* Malassezia* spp. isolated from mothers in this study was different from reports from France and Japan, in which *M. sympodialis* and *M. restricta* were the predominant isolates, respectively [ 21 , 22 ]. At the same time, in line with the results of a study performed by Bernieral et al. [ 22 ], *M. globosa* was the most typical spp. in the present study.

A comparative molecular approach in* Malassezia* isolated from neonates and their mothers could be able to elucidate the role of the mother in the colonization of* Malassezia* in neonates. The results showed a high rate of similarity (76%) in* Malassezia* spp. colonization of mothers and neonates during the first week after birth. These results are in line with those of an experiment carried out by Bernier et al., who reported 60% similarity between spp. isolated from mothers and neonates [ 22 ]. At the same time, isolates typing is required for complete assurance, which was not one of the achievable goals in this study. One of the reasons for the increase of this similarity in the present study was the emphasis on performing kangaroo mother care in the early hours of birth nowadays, compared to the past [ 23 ]. 

According to the findings, the frequency of *M. globosa* spp. (the most isolated spp. in both populations) was higher in the mothers than the neonates (67.4% versus 60.9%). Differences in sebum secretion rate and sebum composition during pregnancy could be considered a reasonable explanation for the different growth patterns of* Malassezia* spp. in the aforementioned population [ 24 ].

We analyzed most of the main demographic variables associated with colonization using Fisher’s exact or chi-square tests. In some earlier studies, no significant relationship was found between neonates who were or were not breastfed in terms of* Malassezia* colonization [ 14 , 18 ]. However, according to our statistical analysis, breastfeeding contributed to the positive culture results. Breastfeeding is encouraged in most NICUs since it provides close contact between the mother and neonate. Therefore, it was assumed to be a risk factor correlated with colonization. The simultaneous colonization of several spp. of* Malassezia* in one person is possible and some isolates of them will be missed in the cultivation process. Therefore, it was better to make the molecular identification directly from the clinical sample instead of the colony.

## Conclusion

The findings support the hypothesis that* Malassezia* colonization develops after the first day of life. Moreover, the colonization of* Malassezia* in neonates is significantly influenced by that of their mothers, which may be associated with breastfeeding.

## Acknowledgments

This study was derived from a thesis submitted in partial fulfillment of the degree of Master’s by Maryam Naderibeni and funded by the Research and Technology Deputy of Shiraz University of Medical Sciences, Shiraz, Iran (grant No. 18245). The authors would like to thank Miss Sima Maher and the Research Consultation Center (RCC) of Shiraz University of Medical Sciences for native English editing of the article.

## Authors’ contribution

K. Z. has contributed to all parts of the project, including writing the paper. H. M., M. Kh., H. Kh., and K. P. have helped in analyzing the data and writing the paper. Furthermore, M. N., M. R.N., M.S., and M.M. managed the samples and laboratory techniques and M. M. supervised all parts of the project and writing the paper.

## Conflicts of interest 

There are no conflicts of interest related to this study.

## Financial disclosure

There are no financial conflicts of interest to disclose.
